# Muva physical activity intervention to improve social functioning in people with a severe mental illness: study protocol of a pragmatic stepped wedge cluster randomized trial

**DOI:** 10.1186/s12888-022-04321-3

**Published:** 2022-11-11

**Authors:** Lisanne Elisabeth Maria Koomen, Ilona Hendrika Theodora van de Meent, Jeroen Deenik, Edwin van Dellen, Hugo Gerard Schnack, Henri van Werkhoven, Wilma Elisabeth Swildens, Berno van Meijel, Wouter Staal, Frederike Jörg, Floortje Scheepers, Wiepke Cahn

**Affiliations:** 1 Utrecht Brain Center, UMC Utrecht, Utrecht, The Netherlands; 2 Lister, Utrecht, The Netherlands; 3 Utrecht University, Utrecht, The Netherlands; 4 Maastricht University, Maastricht, The Netherlands; 5 GGz Centraal, Amersfoort, The Netherlands; 6 Altrecht Mental Health Institute, Utrecht, The Netherlands; 7 Inholland University of Applied Sciences, Amsterdam, The Netherlands; 8 Amsterdam UMC (VUmc), Amsterdam Public Health Research Institute, Amsterdam, The Netherlands; 9 Parnassia Psychiatric Institute, The Hague, The Netherlands; 10 University Leiden, Leiden, The Netherlands; 11 RadboudUMC, Nijmegen, The Netherlands; 12 University Medical Center Groningen, Groningen, The Netherlands

**Keywords:** Severe mental illness, Social functioning, Physical activity, Healthy lifestyle, Supported housing, Implementation

## Abstract

**Background:**

People with severe mental illness (SMI) often suffer from long-lasting symptoms that negatively influence their social functioning, their ability to live a meaningful life, and participation in society. Interventions aimed at increasing physical activity can improve social functioning, but people with SMI experience multiple barriers to becoming physically active. Besides, the implementation of physical activity interventions in day-to-day practice is difficult. In this study, we aim to evaluate the effectiveness and implementation of a physical activity intervention to improve social functioning, mental and physical health.

**Methods:**

In this pragmatic stepped wedge cluster randomized controlled trial we aim to include 100 people with SMI and their mental health workers from a supported housing organization. The intervention focuses on increasing physical activity by implementing group sports activities, active guidance meetings, and a serious game to set physical activity goals. We aim to decrease barriers to physical activity through active involvement of the mental health workers, lifestyle courses, and a medication review. Participating locations will be divided into four clusters and randomization will decide the start of the intervention. The primary outcome is social functioning. Secondary outcomes are quality of life, symptom severity, physical activity, cardiometabolic risk factors, cardiorespiratory fitness, and movement disturbances with specific attention to postural adjustment and movement sequencing in gait. In addition, we will assess the implementation by conducting semi-structured interviews with location managers and mental health workers and analyze them by direct content analysis.

**Discussion:**

This trial is innovative since it aims to improve social functioning in people with SMI through a physical activity intervention which aims to lower barriers to becoming physically active in a real-life setting. The strength of this trial is that we will also evaluate the implementation of the intervention. Limitations of this study are the risk of poor implementation of the intervention, and bias due to the inclusion of a medication review in the intervention that might impact outcomes.

**Trial registration:**

This trial was registered prospectively in The Netherlands Trial Register (NTR) as NTR NL9163 on December 20, 2020. As the The Netherlands Trial Register is no longer available, the trial can now be found in the International Clinical Trial Registry Platform via: https://trialsearch.who.int/Trial2.aspx?TrialID=NL9163.

## Background

People with severe mental illness (SMI) often suffer from long-lasting psychiatric symptoms, despite treatment according to current guidelines [1, 2]. These symptoms negatively influence their social functioning, their ability to live a meaningful life and their participation in society [3]. Moreover, people with SMI experience difficulties to connect with other people, and to enjoy their leisure time [3, 4]. This substantially affects their quality of life [1, 2, 5]. An increase in physical activity appears to improve social functioning and quality of life by decreasing psychiatric symptoms [6–8]. Increasing physical activity can also improve the physical health of people with SMI [8, 9]. This is important since people with SMI on average have a 15–20 years lower life expectancy compared to the general population [10–14], which is to a considerable extend due to diseases in which an inactive lifestyle plays a role [10, 11, 15–17]. Therefore, interventions to promote physical activity are essential in the treatment of people with SMI to improve social functioning.

However, people with SMI experience many barriers to increase physical activity. First, the psychiatric illness and symptoms, such as positive and negative symptoms, loss of initiative, depressed mood, fatigue, anxiety, insomnia, and problems in memory tasks and executive functions, form a barrier [18, 19]. Second, the treatment of their psychiatric illness can be a barrier. Psychotropic drugs can cause weight gain, sedation, fatigue, secondary negative symptoms, and movement disturbances as extrapyramidal symptoms which hamper an active lifestyle [18]. Third, a bad physical condition caused by high smoking rates [20] and unhealthy nutritional habits [21] forms a barrier to becoming physically active [18]. Fourth, physical activity and physical wellbeing do not have priority in the treatment since treatment often focusses on psychiatric symptoms and psychosocial problems [22]. Fifth, people with SMI experience barriers related to psychological factors such as low self-esteem, self-stigmatization, experienced stress, and feeling socially unsafe. Sixth, societal barriers such as stigma, lack of social support, and lack of financial resources form barriers [18, 19, 23, 24].

To improve social functioning by promoting physical activity in the treatment and daily life of people with SMI, it is important to lower these barriers. Mental health workers may have an important role in overcoming these barriers. They are responsible for the personal guidance in the daily living environment of people with SMI, for example, those receiving support from a supported housing organization. These workers are in close contact with the patient and could facilitate a supportive environment for making healthy lifestyle choices [25], set a good example, motivate the patient, provide social support, help with planning physical activities, and might thus potentially be of great value in overcoming barriers and to help patients with becoming physically more active. Previous research [26] showed that a lifestyle intervention including staff participation indeed improved physical activity and social functioning in hospitalized patients with SMI.

Moreover, the lifestyle and personality of the health care worker might play a role in their capacity to help patients overcome barriers to physical activity. Previous research in general health care showed that healthier lifestyle habits of the health care professional are associated with lifestyle counseling and referring practices to lifestyle interventions [27–30]. In addition, it was found that mental health workers, who worked in a supported housing organization, achieved the best psychiatric rehabilitation outcomes such as self-reliance in patients when they scored high on the personality trait ‘conscientiousness’ [31]. The authors suggested that conscious care planning is essential for the recovery process and that the mental health worker plays a key role in the recovery process [31]. However, it remains unknown if the lifestyle and personality type of the mental health worker matters for promoting physical activity in people with SMI.

eHealth and gamification may offer support in overcoming barriers and motivating people with SMI in becoming physically more active [32]. In the past decade, the effect of eHealth on physical activity has been increasingly investigated [33, 34]. Research shows that eHealth could increase physical activity in the general population since it can help with goal setting, monitoring, and achieving goals by sending reminders, educating, and rewarding physical activity [33, 34]. There is growing interest in the application of gamification in eHealth since games can stimulate and motivate physical activity in a playful way [35]. eHealth is also feasible for people with SMI [36], but research is limited on the effects of eHealth and gamification on physical activity [32].

Not only do people with SMI experience barriers with physical activity interventions, but the implementation of physical activity interventions in day-to-day practice is also difficult [37, 38]. A study by Deenik et al. [38] in an inpatient setting showed several barriers at the organizational level such as lack of time and financial resources. However, evidence on implementation barriers and facilitators of physical activity interventions is limited, and studies [6, 7, 37] highlight the need for more knowledge on implementation factors of physical activity interventions in day-to-day practice.

## Objectives

We designed the Muva physical activity intervention for people with SMI to improve their social functioning. The intervention focuses on increasing physical activity by implementing group sports activities, active weekly meetings with mental health workers, and a serious game to set physical activity goals. The intervention aims to decrease barriers to physical activity through active involvement of the mental health worker, supporting lifestyle courses, and a medication review.

We will investigate if social functioning and mental and physical health of patients can improve through this package of interventions. We hypothesize that social functioning and quality of life will improve, psychiatric symptoms will decrease, and physical health will improve with an increase in cardiorespiratory fitness, and a decrease in movement disturbances and cardiometabolic disease risk factors.

To gain understanding on movement disturbance severity and the impact on motor execution in SMI, the Sint Hans Rating Scale and gait analysis with markerless motion capture will be performed during the physical measurements in the UMCU. The obtained results may provide new information on which specific movement interventions are recommendable for a better and sustainable implementation of physical activities for people with SMI in the future.

Moreover, we will examine if the lifestyle habits and/or personality type of the mental health worker are associated with the outcomes. Our hypothesis is that mental health workers with healthier lifestyles and a conscious personality type can contribute more to an active and healthy lifestyle of their patients since they might value physical activity as important and are able to consciously plan physical activities and oversee barriers for patients. In addition, we aim to evaluate the barriers and facilitators of the implementation of the intervention by conducting semi-structured interviews with location managers and mental health workers.

This paper aims to describe the Muva study in detail according to the SPIRIT statement [39].

### Methods

#### Study design and setting

This study is a pragmatic stepped wedge cluster randomized controlled trial that will be conducted at supported housing organization Lister and the University Medical Centre Utrecht (UMCU) in the Netherlands. This design allows us to implement the intervention sequentially in the participating teams and to study the effects of the Muva intervention.

A supported housing organization is an organization that supports people with SMI in their rehabilitation by giving assistance in several domains in patients’ lives such as housing, daily activities, (voluntary) work, building a social network and finances in order to promote independency. Within this setting, they do not provide psychiatric treatment, this is provided by the general practitioner or a psychiatrist from another organization. Patients have a key mental health worker, with a mental health degree of a university in applied sciences, who is responsible for the guidance plan and the rehabilitation support. This worker has guidance meetings with the patient for at least once a week, but more frequently if the condition of the patient requires more support. The patient population of Lister is diverse with patients living independently and receiving ambulatory care, patients who live in small assisted living facilitations and patients who live in a (group) house with 24 h care.

All teams of the supported housing organization (*n* = 30) will be invited to participate in the study. Teams in four different areas of Utrecht will be chosen to participate to prevent spillover effects and contamination since teams work closely together in these areas. Every area will form a cluster. Three clusters will start the intervention in a stepped wedged order, within intervals of three months, and one cluster will remain the control group during the study period (Table 1). To determine which area is allocated to which cluster to start the intervention, a simple randomization method is performed by drawing lots. Hence, randomization is done at cluster level and not at patient level. Randomization at patient level is not possible since teams deliver equal rehabilitation care to the patients of their team. Given the stepped wedge design and the vulnerable patient group, for whom continuity of research team is desired, participants and the research team know when each cluster will crossover from unexposed to the exposed period, also reducing the possibility of drop-out. Nevertheless, to reduce rater bias the final measurement at the 21th month of the study, will be performed blind to the intervention exposure duration (Table 1).

**Table 1:**
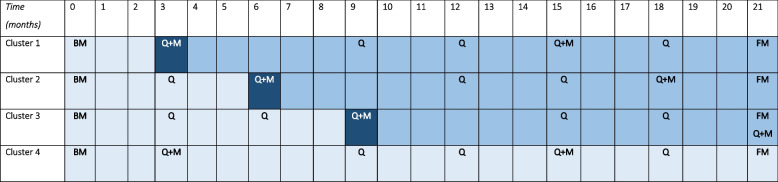
Design of the pragmatic stepped wedge cluster randomized controlled trial. Control period marked light blue, transition period marked dark blue, and intervention period marked intermediated blue. Time in months. BM = baseline measurement FM = Final measurements with questionnaires, performed at the UMCU. Q = online questionnaire Q + M = measurement with questionnaires and physical measurements, performed at the UMCU

To evaluate changes in social functioning, and mental and physical health patients will receive a series of four online surveys. They will be invited before and after the intervention period to the UMCU for the surveys and physical measurements and a third time for only the surveys at the final measurement at the 21th month of the study. Mental health workers will receive one online questionnaire at baseline (Table 1).

### Study population

We will recruit 100 patients and their mental health workers. All participants will be informed about the study and asked for consent. Patients are included if they are ≥ 16 years old, diagnosed with a severe mental illness (i.e., a psychiatric disorder classified by the DSM-5 causing serious limitations in psychosocial functioning for a duration of ≥ 2 years), and can give informed consent and read and speak Dutch. Patients are not eligible for inclusion if their psychiatric condition hinders informed consent or/and participation in the intervention according to their mental health workers.

Mental health workers are included when they can give informed consent and read and speak Dutch.

#### Muva intervention

As summarized in Table 2, the Muva intervention is a physical activity intervention that aims to improve social functioning by promoting physical activity in patients and their mental health workers by focusing on activities to become physically more active and activities to lower barriers to physical activity for patients with SMI.

**Table 2:** Overview of the Muva intervention

Phase	Activities	Targeted at
Step 1: Evaluating physical activity and goal setting	• Physical activity check to: ◦ Evaluate amount of physical activity ◦ Assess barriers and opportunities ◦ Set goals • Serious game ‘Muva’ for goal setting, reminders and motivation	• Patients • Mental health workers
Step 2: Participating in physical activity activities, lifestyle courses, and medication review	• Group sports activities: e.g., group walks, yoga, boxing, fitness, football • Guidance meetings will be done for at least once a week in an active way, e.g., during a walk or a bike ride • Lifestyle courses: a. Healthy food program: A five-week course in which participants learn the basics of healthy food and try to improve their eating habits b. Improve sleeping program: A 6-week insomnia cognitive behavioral therapy course in which participants learn to improve their sleep quality c. Smoking cessation program: An 8-week course with elements of cognitive behavioral therapy in which participants do an attempt to quit smoking d. Individualized food advice: Dietary advice by filling in the Dutch Healthy Diet Index, a questionnaire based on the nutrition advice of the Dutch center for nutrition • Medication review	• Patients • Mental health workers • Patients • Mental health workers • Patients

Each patient and each mental health worker start with filling in a physical activity check together to evaluate physical activity, barriers to increase physical activity and set goals with the Muva serious game. The Muva serious game is an app that is specially designed for people with SMI and their mental health worker. It helps users to formulate small daily goals to increase physical activity. It gives notifications at desired times to remind the user of the formulated goals and planned activities. Users can note in the app whether they have reached their goal and how their mood was on a particular day. They will receive rewarding elements and points when they open the app and report in the app if they have reached their goal. In the weekly appointments with their key mental health worker, they will open the app and discuss their progress.

The activities to become physically more active are 1) group sports activities and 2) weekly active guidance meetings with the mental health worker. The activities to lower barriers to becoming physically active are 1) active involvement of the mental health worker, 2) lifestyle courses aimed at smoking cessation, improving sleep and healthy eating and 3) a medication review.

Since patients often use psychotropic medication that might cause barriers for increasing physical activity, a medication review will be performed in current practice. A regular medication review should be a part of care as usual but is not systematically performed and many patients keep the same medication for a long period. The medication review will be done at the beginning of the intervention period in a multidisciplinary team meeting with a psychiatrist, a psychiatric trainee, and a pharmacist. Before this meeting information on psychiatric health and medical history will be retrieved from the psychiatrist or general practitioner of the patient and a structured medication questionnaire will be conducted. Medication advice will be based on current guidelines. The research team will discuss the medication advice with the psychiatrist and/or general practitioner of the patient and they will discuss the advice with the patient.

The Muva intervention is targeted at all patients from the participating locations and their mental health workers, not only for the patients and workers who participate in this study, since the explicit aim is to implement the Muva intervention into the routine care of the supported housing organization.

Before the intervention period, an implementation scan will be performed in which we will ask the locations what type of physical activity and lifestyle-related activities they already carry out, what facilities for activities they have, and which staff members can be involved with the intervention. The Muva intervention will then be adjusted to the specific location to lower implementation barriers.

The intervention starts with a transition period (Table 1) in which the intervention is introduced, and all workers are trained on how to discuss physical activity with their patients. All participating teams will assign a dedicated mental health worker who manages and implements the intervention with the research team's assistance in collaboration with a coordinator from the supported housing organization. The dedicated mental health worker will receive training from the research team and will have monthly meetings with the research team to discuss the implementation process. The lifestyle courses will be given by two mental health workers in every team who will be trained to give these programs.

#### Care as usual

During the control phase, patients receive care as usual. Care as usual consists of rehabilitation care focused on housing, (voluntary) work, and supporting patients to live independently. To stimulate an active control condition during the study period, there will be a general healthy lifestyle campaign at the supported housing organization that also targets the control group. In this campaign, several news messages on a healthy lifestyle will be posted on the organizations’ website targeting the mental health workers, but there will be no specific physical activity intervention targeting the patients. Next to the care from the supported housing organization, patients receive psychiatric care from their general practitioner or psychiatrist. This psychiatric care can consist of pharmacological treatment, psychotherapy, and a yearly somatic screening and medication review.

#### COVID restrictions

When mandatory government measures will not allow group meetings, the group courses will be done online via live videoconference meetings.

## Outcomes and measurements

### Patients

#### Primary outcome

The primary outcome is social functioning as measured by the Social Functioning Scale (SFS) [40]. The SFS consists of seven domains of which we will use the domains social withdrawal, interpersonal communication, independency performance, independency competence, and recreation. We will not use the domains social interaction and work because due to the COVID-19 pandemic restrictions and lockdowns options for day programs (work) and social interaction in restaurants, cinemas and nightlife might be limited.

#### Secondary outcomes

##### Physical activity

The International Physical Activity Questionnaire Short Form (IPAQ-SF) will be used to measure physical activity. The IPAQ-SF consist of seven questions on vigorous, moderate physical activity, walking, and sitting during the last seven days [41].

##### Symptom severity

Symptom severity will be measured by the Brief Symptom Inventory (BSI). The BSI consists of 53 items divided into nine symptom dimensions. The number of symptoms present per symptom dimension will be used [42].

##### Quality of life

The WHOQoL-BREF will be used to measure quality of life. The WHOQoL-BREF consists of 24 items divided into the domains physical health, psychological, social relationships, and environment and two general health items. [43].

##### Cardiorespiratory fitness

Cardiorespiratory fitness will be measured with a VO_2_ max assessment with the Åstrand Bike Test. The submaximal Åstrand Bike Test is considered a low invasive VO_2_ max test because of its duration of 6 min and people do not need to perform at maximum capacity [44, 45].

##### Postural adjustment and movement sequencing in gait

A real-time video camera and markerless motion capture camera’s is set up to measure postural adjustments and movement sequencing in gait. The technological application of markerless motion capture during the gait analysis supports the validation in calculating body proportions (angles between body parts), timing, direction, and velocity without attaching markers to the skin. The main items of observation are; Posture, movement initiation and movement sequencing [46–49].

##### Movement disturbances

The Sint Hans Rating Scale will be also used to measure movement disturbances. The Sint Hans Rating Scale is an evaluation tool for akathisia, dyskinesia, dystonia, and Parkinsonism. It consists of 29 items divided into four dimensions of movement disturbances [50].

##### Cardiometabolic disease risk factors

Blood pressure, waist circumference, and BMI are measured during the measurement in the UMCU. The Dutch guideline on somatic screening for people with SMI recommends doing a yearly somatic screening including a blood test to test HbA1c, total cholesterol, LDL-cholesterol, HDL-cholesterol, and triglycerides. The results of this blood test will be retrieved from the psychiatrist or general practitioner when patients give permission.

Attendance at intervention group activities will be monitored.

### Mental health worker

#### Primary outcome

##### Physical activity and lifestyle habits

The primary outcome for the mental health worker is their physical activity and lifestyle habits. The IPAQ-SF and the Dutch Healthy Diet Index will be conducted. The Dutch Healthy Diet Index is a screener for dietary quality based on Dutch and international nutrition guidelines and assesses 15 components of the Dutch dietary guidelines [51]. They will also be asked for their smoking status, drinking habits, length, and weight.

#### Secondary outcome

##### Personality traits

The personality traits will be measured with the NEO five-factor inventory. The NEO five-factor inventory is a 60-item questionnaire that measures the five domains of personality neuroticism, extraversion, openness, agreeableness, and conscientiousness [52].

#### Implementation barriers and facilitators

At the end of the intervention period, we will conduct semi-structured interviews on the implementation of the intervention with the location managers and dedicated mental health workers. Questions on the content of the intervention, barriers and facilitators during the implementation process, and perceived outcomes of the intervention will be asked. Interviews will be recorded, transcribed, and analyzed by direct content analysis by two researchers independently. We will also evaluate the implementation by monitoring which sports activities are provided, what the frequency of the lifestyle courses is and we will assess the percentage of patients in which physical activity goals are part of the guidance plan.

### Measurements

Table 1 provides an overview of the scheduling of the measurements and Table 3 shows the content of the measurements. The online questionnaire always consists of the SFS. To lower the burden on patients and to increase the reliability of the answers we will include the WHOQOL-BREF or the BSI in the online questionnaire in alternating order. This order will be decided based on randomization. The mental health worker will receive one online questionnaire.

**Table 3:** Overview of measurements

**Patient**	
**Baseline measurement (T = 0)**	Sex, age, diagnosis, form of supported housing, smoking and drinking habits **Questionnaires**: SFS, BSI/WHOQOL-BREF
**Measurements at the UMCU (In the intervention groups: prior to the start of intervention and 12 months after the start of intervention)**	Medication use, smoking, and drinking habits **Questionnaires**: SFS, BSI, WHOQoL-BREF, IPAQ-SF, Sint Hans Rating Scale, structured questionnaire to indicate physical complaints that may influence gait, structured medication questionnaire **Measurements**: VO_2_ max Åstrand Bike test (submaximal), posture and gait analysis with a real-time video camera and markerless motion capture, blood pressure, length, weight, waist circumference Blood tests retrieved from medical records: HbA1c, total cholesterol, LDL-cholesterol, HDL-cholesterol, triglycerides
**Online questionnaire measurement (T = 3, 6, 9, 12, 15, 18, 21)**	**Questionnaires**: SFS, BSI/WHOQOL-BREF
**Mental health worker**	
**Baseline measurement (T = 0)**	Sex, age, length, weight, smoking and drinking habits **Questionnaires**: IPAQ-SF, Dutch Healthy Diet Index, NEO five-factor inventory

### Research procedures

This study will be conducted according to the principles of the Declaration of Helsinki (amended version in October 2013) and following Good Clinical Practice guidelines from the European Medicines Agency (ICH E6, R2) and the Medical Research Involving Human Subjects Act (WMO).

### Recruitment

Patients will be recruited via their key mental health worker. Mental health workers will receive instructions and flyers about the study. During the recruitment phase and baseline measurement patients will not get detailed information on the elements of the Muva intervention, but are broadly informed that the study is about examining and improving the mental and physical health of people with SMI. This is done to limit selection bias since patients that are interested in physical activity might be more likely to participate in a study on increasing psychical activity. After the patient agrees to participate, the patient’s key mental health worker will also be recruited. Patients will receive two vouchers of in total of 50 euros for their participation to reimburse time and travel costs.

### Statistical analyses

#### Sample size

The sample size is calculated for the primary outcome social functioning. For the SFS we assumed an intra-cluster correlation of 0.1, a between-level correlation of 0.8, and a standard deviation of 9 [34, 41]. The anticipated dropout rate is 30%, as physical activity interventions in mental health care have average dropout rates of 26.7% [19]. We performed simulations to determine the power of the trial design to demonstrate an increase of at least 3 points after one year in the SFS. With measurements on seven time points in 100 patients, the study will have a power of 0.93.

### Data analysis

The primary analysis in this study evaluates if the Muva intervention improves social functioning in comparison to care as usual. This will be analyzed with a mixed-effects linear regression model taking into account clustering of outcome measurements within individuals and centers using random effects and possible confounding factors age, gender and diagnostic subgroups using fixed effects. In addition, we will include lifestyle habits and character traits of the mental health worker as dependent variables to analyze the association between the mental health workers’ characteristics and the outcome social functioning.

Modified intention-to-treat analysis will be done, but if patients move to a team of the supported housing organization without the Muva intervention or rehabilitation care stops within 3 months of intervention, patients will be analyzed in the control group since they will no longer receive the Muva intervention. Patients that drop out from the study, will be analyzed in the group they participated in.

Sensitivity analysis will be performed on cluster, attendance at intervention activities, and medication changes. Dropout analysis will be performed. Analysis will be done in IBM SPSS statistics 25 and R version 4.0.4, using a 95% confidence interval (*p* < 0.05).

### Data management

All data will be saved in a secured online database of the UMCU in electronic case record forms. Data will only be accessible to the research team associated with the MUVA study through two-way factor authorization. The acquired data will be used without traceable personal data for scientific analysis. Data will be stored for 15 years within the UMCU.

### Involvement of experienced experts

This study protocol was designed in collaboration with a group of experts by experience. They will be involved in the entire study process to adjust the intervention to the patient’s needs and to evaluate the study results for recommendations for clinical care. We have recruited experienced experts from Lister, patient organizations, and via social media.

## Discussion

This trial is innovative since it aims to improve social functioning by promoting physical activity by focusing on lowering barriers for people with SMI to becoming physically active through the active involvement of the mental health worker, supporting lifestyle courses, and a medication review. Through this strategy, we aim to increase participation and motivation. Second, we will use the Muva serious game that is specifically designed for people with SMI and their mental health workers to support physical activity goals setting and to motivate participants to reach their goals. Third, the intervention will not only target the patient but also the mental health worker since they are an important factor in the environment of the patient and could facilitate a supportive environment for becoming physically active. We hypothesize that key workers with healthier lifestyles and a conscious personality type can contribute more to an active lifestyle of their patients and we will analyze if there is indeed a correlation. Fourth, a unique element of this trial is that we will investigate the posture and movement of the patient extensively with movement sequencing in gait with a real-time video camera and marker less motion capture and the Sint Hans Rating Scale to research movement disturbances that can form barriers to start and continue exercising and form risks for injuries for people with SMI. Fifth, we will investigate implementation barriers and facilitators and will contribute to the urgently needed evidence on translating evidence from physical activity intervention randomized controlled trials into real-life practice [6, 7, 37]. With these strategies, we will complement previous knowledge on physical activity interventions in people with SMI.

This study also has limitations. First, there is a risk of poor implementation of the intervention which will hamper the outcomes. Therefore, we will monitor the intervention activities at all locations. Second, we will risk missing data since not all patients will be able to participate in the measurements at the UMCU because their symptoms will form a barrier to coming to the hospital. However, since this is a pragmatic trial in real-life practice, we will include these patients, also to minimize selection bias. In addition, blood tests will not be performed in the study, but the results will be retrieved from the psychiatrist or general practitioner when patients give permission. Third, we will perform a medication review in the intervention group to optimize medication and decrease side effects that might form barriers to physical activity. This is part of care-as-usual, but medication reviews are not always performed. This can cause bias since outcomes might be influenced by medication changes and not by increased physical activity. We will perform sensitivity analysis on medication changes to evaluate this possible bias.

In conclusion, we hope that this study will provide knowledge on the effectiveness of physical activity interventions on social functioning, and mental and physical health for people with SMI, will give more insight into the role of the mental health worker in physical activity interventions in mental health care and will contribute to the evidence on the implementation of physical activity interventions in real-life practice.

## Data Availability

Not applicable.

## Abbreviations

BSI: Brief Symptom Inventory
IPAQ-SF: International Physical Activity Questionnaire Short Form
SFS: Social Functioning Scale
SMI: Severe mental illness
UMCU: University Medical Centre Utrecht
